# Emergency general surgeons: the special forces of general surgery (the “navy seals paradigm”)

**DOI:** 10.1186/s13017-020-0293-7

**Published:** 2020-02-12

**Authors:** Fausto Catena, Walter Biffl, Belinda De Simone, Massimo Sartelli, Salomone Di Saverio, Yoram Kluger, Ernest E. Moore, Luca Ansaloni, Federico Coccolini

**Affiliations:** 1 Department of Emergency and Trauma Surgery, University Hospital of Parma, Parma, Italy; 2 Trauma and Acute Care Surgery, Scripps Memorial Hospital, La Jolla, San Diego, CA USA; 3 Department of General, Emergency, Metabolic Surgery, Centre Hospitalier Intercommunal Poissy/Saint-Germain-en-Laye, Poissy, France; 4 Department of General Surgery, Macerata Hospital, Macerata, Italy; 5 Cambridge University Hospitals NHS Foundation Trust, Cambridge, UK; 6 Division of General Surgery, Rambam Health Care Campus, Haifa, Israel; 7 Ernest E Moore Shock Trauma Center at Denver Health and University of Colorado, Denver, Colorado USA; 8 Department of Emergency and Trauma Surgery, Bufalini Hospital, Cesena, Italy; 9 General, Emergency and Trauma Surgery Dept, Pisa University Hospital, Pisa, Italy

**Keywords:** Emergency surgery, General surgery, Health system, Organizational solution, Surgical training, Academic education

## Abstract

Emergency surgeons have a crucial role in bridging the gap of skills resulting from the well-known general surgery fragmentation. The multi-specialist general surgery approach is still necessary to define proper diagnosis and therapy priorities in an emergency. Governments have to find effective organizational solutions to maintain emergency general surgery standards of care and to improve them further.

The future of general surgery and emergency general surgery (EGS) is still debated. Over the past two decades, the specialty of general surgery has become increasingly fragmented. Many patients have benefitted from the focus and expertise of hepato-pancreato-biliary, colorectal, breast, and endocrine surgeons. On the other hand, as specialists, surgeons have lost their ancestral general surgeon’s eclecticism [1]. A clear example is vascular surgery: this was part of the general surgery domain but developed to a well-recognized autonomous specialty; consequently, a vascular surgeon is not able to perform emergency general surgery anymore. Thus, in the near future, we will encounter “a new species” of subspecialist general surgeons with evident gaps in skills that may be incapable to deal with all diverse emergency surgery cases. Paradoxically, in this case, Darwin’s natural selection law was not followed: general surgeons’ species evolutionary curve was in contrast with environmental acute surgical patients’ demands.

On the other side, there are unchanged needs from acute surgical patients which are even more challenging with the increasing elderly. Prompt and right diagnosis and treatments in various advanced general conditions are essential and lifesaving. Recently, an emergency laparotomy audit in the UK found a 20% mortality after emergency laparotomies performed during night shifts [2].

This means that the multi-specialist general surgery approach is still necessary to define proper diagnosis and therapy priorities in an emergency. The rise of EGS as a specialty is an answer to this necessity as well as the recognition that the acuity of the patients admitted for acute surgical diseases is unique and deserves special attention.

As the footprint of EGS expands, this issue takes on enormous importance. A recent analysis in the USA found that 20% of the inpatient population comprises patients with trauma or EGS diagnoses, and they are responsible for 25% of inpatient costs [3]. Health care utilization rates are higher, and hospital stays are longer for these patients. The growing population of elderly patients, with their specialized needs, magnifies the problem.

The challenge confronting surgeons, healthcare systems, and governments is to meet the needs of the patients and the system [4]. While we debate the merits of regionalization of EGS services, the front lines of all hospitals must be manned by appropriately trained surgeons.

Governments have to find effective organizational solutions to maintain EGS standards of care and to improve them further avoiding national health systems failures. This will require EGS services and critical surgical emergency centralization to provide optimal patient care as well as education.

The “navy seals concept” is the paradigm of this solution.

Special forces are military units trained to conduct special operations in difficult environments.

NATO has defined special operations as “military activities conducted by specially designated, organized, trained, and equipped forces, manned with selected personnel.”

Depending on the country, special forces may perform different functions including airborne operations, counter-insurgency, counter-terrorism, foreign internal defense, covert operations, direct action, hostage rescue, high-value targets/manhunting, intelligence operations, mobility operations, and unconventional warfare.

The main characteristics of an emergency general surgeon include [5–7]:

- Availability 24/24 h, 7/7 days
- Capability to manage difficult situations
- Familiarity with different scenarios including war surgery
- Competency in mass casualties
- Capability to rescue acute care patients (major general surgery complications)
- Ability to perform aggressive surgical approaches when required
- Ability to tailor surgical approaches
- Familiarity with new and emerging technology (hybrid rooms, endovascular techniques, stents, REBOA, shunts, ECMO, etc.)
- Conditioning based on daily surgical activity, with particular resistance to stress and fatigue
- Ability to problem solve and innovate
- Ability to interact and communicate with all surgical subspecialties (including vascular and thoracic)
- Knowledge and understanding of different patient physiological changes
- Ability to effectively communicate with patients and relatives avoiding legal conflicts

It is evident from this list that emergency general surgeons are a sort of special force of general surgery: they are selected personnel who have to be preserved.

Moreover, they own interdepartmental and interdivisional connections, having to manage patients throughout the hospital. Moving throughout the hospital, acute care surgeons are trained to understand different situations linked to the different clinical scenarios and to understand medical and physio-pathological reasons for the different specializations. They are the most specialized among the generalists and the most general among the surgeons. Moreover, they have an important role in maintaining specialties coordination when facing surgical or medical complications.

In their daily activity, they represent the hospital in its most important role: dealing with urgencies, emergencies, and all critical situations.

Patients and families can find a valid counterpart on the hospital side capable to present emergency surgeons as the only representative of the hospital, who are able to explain and to solve problems whenever possible. Whenever not possible, emergency general surgeons are able to communicate with patients and relatives in a professional way.

National governments must understand that police services need special forces as much as national health systems need acute care surgery teams.

It is mandatory to organize a National Emergency General Surgery Force that involves all national hub centers to guarantee a prompt and standardized acute care surgery response in case of usual daily activity or in case of mass casualties incidents.

This must be achieved with specific acts finalized to preserve population health in case of various surgical emergencies.

It is not an overestimation of standard work; it is an effective and organized approach to the acute setting as they are civil protection departments.

This is a scientific public plea to all world’s national presidents and prime ministers, written by world experts in emergency general surgery: this is the only “road-map” to avoid undertreatments in emergency surgery as it always means more morbidity and more mortality.

## Data Availability

Not applicable

## Abbreviations

ECMO: Extracorporeal membrane oxygenation
EGS: Emergency general surgeon(s)
NATO: North Atlantic Treaty Organization
REBOA: Resuscitative endovascular balloon occlusion of the aorta
